# Seven genome sequences of *Rothia* spp. isolated from human saliva

**DOI:** 10.1128/mra.00465-25

**Published:** 2025-07-07

**Authors:** Allen Choi, Lindsey Pia, Justin R. Kaspar

**Affiliations:** 1 Division of Biosciences, The Ohio State University College of Dentistry, Columbus, Ohio, USA; Portland State University, Portland, Oregon, USA

**Keywords:** oral microbiology, oral microbiome, *Rothia*, respiratory pathogens, genomes

## Abstract

*Rothia* are understudied commensal bacteria within the human oral cavity and respiratory tract and can cause opportunistic infections. We report the complete genome sequences of seven *Rothia* spp. strains isolated from pooled human saliva, including four strains of *Rothia mucilaginosa* (59.5% GC) and three strains of *Rothia dentocariosa* (53.8% GC).

## ANNOUNCEMENT

Three species of the genus *Rothia* are known to colonize human hosts: *Rothia aeria*, *Rothia dentocariosa*, and *Rothia mucilaginosa*. For all three species, fewer than 30 high-quality genomes are publicly available (1). All *Rothia* species are gram-positive, facultative anaerobes with pleomorphic morphologies (2, 3). *Rothia* spp. colonizing humans are commonly found in the oral cavity and respiratory tract and are considered to be commensal bacteria that can cause opportunistic infections such as endocarditis, pneumonia, and septicemia (4–6). The role of *R. dentocariosa* in the development of dental caries is unknown, with the abundance of all *Rothia* spp. in the oral cavity decreasing as carious lesions progress (7–10).

Recently, our group isolated over 100 strains from commercially available pooled human saliva (11). This included 11 total isolates of *R. mucilaginosa* and 3 of *R. dentocariosa*. The first four isolated strains of *R. mucilaginosa* and all three *R. dentocariosa* isolates were selected for whole-genome sequencing. Isolates were first resuscitated from a −80°C glycerol stock using a BHI agar plate and grown for 48 h. Afterward, 5 mL of BHI broth was inoculated and grown overnight (37°C and 5% CO_2_). The following morning, genomic DNA was purified with the DNeasy PowerLyzer Microbial Kit (Qiagen; Catalog # 12255-50), and concentration was determined with a Qubit Flex Flurometer (ThermoFisher; Catalog # Q33327) and the Qubit dsDNA BR Assay Kit (ThermoFisher; Catalog # Q32850) according to the supplier’s protocol. The Microbial Genome Sequencing Center performed combined short- and long-read sequencing (Small Nanopore Combo; Illumina and Oxford Nanopore technologies [ONT], respectively) and *de novo* assembly. Default parameters were used except where otherwise noted. Illumina sequencing was performed on an Illumina NovaSeq X Plus, producing 2 × 151 bp pair-ended reads after libraries were prepared with the Illumina DNA Prep kit. No additional DNA fragmentation or size selection steps were performed. Demultiplexing, quality control, and adapter trimming were performed with bcl-convert (version 4.2.4). Nanopore sequencing, prepared using the Oxford Nanopore Technologies (ONT) Ligation Sequencing Kit (SQK-NBD144.24) using the supplier’s instructions, was performed on an Oxford Nanopore MinION Mk1B sequencer. Guppy (version 6.5.7) was used for basecalling, demultiplexing, and adapter removal (12). Total reads and coverage for each isolate sequenced are listed in Table 1. *De novo* genome assemblies were generated from ONT read data with Flye (version 2.9.2) using the nano-hq model (13). Subsequent polishing used the Illumina read data with Pilon (version 1.24) under default parameters (14). Assembled contigs were evaluated for circularization via Circulator (version 1.5.5) using the ONT long reads (15). Assembly annotation was performed by the NCBI Prokaryotic Genome Annotation Pipeline (PGAP) (version 6.9). Finally, assembly statistics were recorded with QUAST (version 5.2.0) (16).

**TABLE 1:** Details of the seven *Rothia* strains sequenced in this study

Strains	*Rothia dentocariosa*			*Rothia mucilaginosa*			
	SOSUI018	SOSUI019	SOSUI028	SOSUI003	SOSUI004	SOSUI007	SOSUI038
BioSample	SAMN45190763	SAMN45190764	SAMN45190765	SAMN45190766	SAMN45190767	SAMN45190768	SAMN45190769
GenBank accession	CP178332	CP178331	CP178330	CP178329	CP178327 CP178328	CP178326	CP178325
SRA accession	SRX28641571; SRX28641582	SRX28641572; SRX28641583	SRX28641577; SRX28641584	SRX28641573; SRX28641578	SRX28641574; SRX28641579	SRX28641575; SRX28641580	SRX28641576; SRX28641581
Total Illumina reads	7,060,312	6,835,362	6,169,240	8,669,170	7,634,164	6,603,002	6,656,362
% bp >Q30	94.2	94.5	94.8	93.6	92.6	94.2	93
Illumina coverage (×)	395	386	350	523	457	402	398
Total Nanopore reads	390,165	387,170	312,465	237,918	259,271	430,003	295,155
% bp >Q20	83.9	84.3	84.6	82.1	78.9	85.1	79.5
*N*_50_ for ONT (bp)	11,583	10,988	13,469	12,746	9,715	12,981	11,374
Nanopore coverage (×)	405	403	382	285	306	523	361
Total assembled contigs	1	1	1	1	2	1	1
Completeness	95.62	95.93	95.93	99.23	98.45	98.88	99.1
Genome size	2,500,955	2,481,985	2,481,987	2,297,340	2,296,472	2,298,761	2,302,922
GC %	53.82	53.88	53.88	59.48	59.55	59.48	59.49
Best match type-strain (% average nucleotide identity)^*a*^	*Rothia dentocariosa* ATCC 17931 (96.57%)	*Rothia dentocariosa* ATCC 17931 (96.7%)	*Rothia dentocariosa* ATCC 17931 (96.7%)	*Rothia mucilaginosa* ATCC 25296 (94.37%)	*Rothia mucilaginosa* ATCC 25296 (94.53%)	*Rothia mucilaginosa* ATCC 25296 (94.37%)	*Rothia mucilaginosa* ATCC 25296 (94.42%)
Annotated genes^*a*^	2,166	2,158	2,152	1,811	1,818	1,814	1,805
Protein coding genes^*a*^	2,091	2,085	2,081	1,740	1,743	1,743	1,732

^*a*^ Number of annotated genes, protein coding genes, and best match type-strain was determined using the NCBI PGAP.

All sequenced strains, except for SOSUI004, were assembled with complete circular genomes. For *R. dentocariosa* genomes, the total length varies between 2,481,985 and 2,500,955 bp, a GC content percentage of 53.8%, and the number of annotated genes between 2,152 and 2,166. For R. *mucilaginosa*, the total genome length varies between 2,296,472 and 2,302,922, with a GC content of 59.5% and between 1,805 and 1,818 annotated genes.

## Data Availability

The genome sequences associated with this work are available under BioProject accession no. PRJNA1194585. The BioSample, GenBank, and Sequence Read Archive (SRA) accession numbers for each isolate are listed in Table 1.
